# Distribution and characteristics of malignant tumours by lung lobe

**DOI:** 10.1186/s12890-024-02918-w

**Published:** 2024-03-04

**Authors:** Yngvar Nilssen, Odd Terje Brustugun, Lars Fjellbirkeland, Åslaug Helland, Bjørn Møller, Sissel Gyrid Freim Wahl, Steinar Solberg

**Affiliations:** 1 Department of Registration, Cancer Registry of Norway, Box 5313 Majorstuen, Oslo, 0304 Norway; 2 Section of Oncology, Vestre Viken Hospital Trust, Drammen, Norway; 3 Institute of Clinical Medicine, University of Oslo, Oslo, Norway; 4 Department of Respiratory Medicine, Oslo University Hospital, Rikshospitalet, Oslo, Norway; 5 Department of Oncology, Oslo University Hospital, Oslo, Norway; 6 Department of Pathology, St. Olavs Hospital, Trondheim University Hospital, Trondheim, Norway; 7 Department of Cardiothoracic Surgery, Oslo University Hospital, Rikshospitalet, Oslo, Norway

**Keywords:** Lung cancer, Lung lobes, Epidemiology, Pathogenesis, Tuberculosis, Pneumoconiosis

## Abstract

**Background:**

The main focus on the characteristics of malignant lung tumours has been the size, position within the lobe, and infiltration into neighbouring structures. The aim of this study was to investigate the distribution and characteristics of malignant tumours between the lung lobes and whether the diagnosis, treatment, and outcome differed based on location.

**Methods:**

This study is based on 10,849 lung cancer patients diagnosed in 2018–2022 with complete data on the location and characteristics of the tumours. The proportions of tumours in each lobe divided by its volume were termed the relative proportion.

**Results:**

The right upper lobe comprised 31.2% of the tumours and 17.6% of the lung volume. The relative proportion of 1.77 was higher than in the other lobes (*p* < 0.001). The right middle lobe had a relative proportion of 0.64 but the highest proportion of neuroendocrine tumours (26.1% vs. 15.3 on average). Surgical resection was more often performed in patients with tumours in the lower lobes, and curative radiotherapy was more often performed in the upper lobes. After adjusting for age, sex, stage, and histology, the location of the tumour was found to be a significant independent predictor for resection but not for survival.

**Conclusion:**

The main finding of the right upper lobe as a site of predilection for lung cancer is similar to tuberculosis and pneumoconiosis. This may be explained that most of the inhaled air, containing bacilli, inorganic particles or tobacco smoke goes to the upper and right parts of the lung.

**Supplementary Information:**

The online version contains supplementary material available at 10.1186/s12890-024-02918-w.

## Introduction

The focus on the macroscopic characteristics of malignant lung tumours has mainly been the diameter, possible tumour invasion of neighbouring structures [1], and whether the tumour location was in the central or peripheral part of the lobe [2]. Less attention has been paid to characteristics as if the tumours and the histological subgroups were evenly distributed in the lung and the possible impact on disease course caused by location.

The significance of the different locations of the tumours was demonstrated by the finding of improved survival in patients with tumours in the upper lobes after curative treatment [3–6]. In one study, Tseng et al. [7] found that 62% of adenocarcinomas (ACs) were in the upper lobes and that mutations of the epidermal growth factor receptor (EGFR) gene were more frequent in ACs in the upper lobes. Increased survival in patients with tumours in the upper lobes [8] has been explained by the higher frequency of EGFR mutations [9] that make these patients eligible for tumour-reducing therapy with tyrosine kinase inhibitors [10].

The five lung lobes have different volumes, and the right lung constitutes 55% and the left 45% of the total lung volume [11–13]. Yamada et al. estimated the relative volumes of the lung lobes in upright position, where the right lung constitutes 53.3% (standard deviation (SD) = 1.3) with the right upper lobe (RUL): 17.6% (SD = 2.3), right middle lobe (RML): 8.5% (SD = 1.4), and right lower lobe (RLL) 27.2% (SD = 2.2). The left lung 46.7% (SD = 1.3), left upper lobe (LUL): 22.4% (SD = 2.0) and left lower lobe (LLL): 24.3% (SD = 2.4) [12].

The aim of the present study was, based on national data to explore the proportion of malignant tumours and their characteristics in the different lobes of the lungs, and their impact on treatment and survival.

## Methods

### Cancer registry of Norway

Since 1952, it has been mandatory for all hospitals, pathology laboratories and general practitioners to report all newly diagnosed malignant diseases to the Cancer Registry of Norway (CRN). The CRN also receives death certificates for all patients with a cancer diagnosis from the Cause of Death Registry. Using the unique, 11-digit personal identification number assigned to all Norwegian citizens since 1964, the CRN is linked monthly with the National Population Register to update vital status (death or emigration), and three times per year with the Norwegian Patient Registry to ensure completeness of cancer cases. All notifications are sent electronically to the CRN. The quality, comparability, completeness, validity, and timeliness of the data in the CRN have been evaluated to be high, with an estimated completeness of 99.2% for lung cancer [14, 15].

### Norwegian lung cancer registry

This quality register for lung cancer within the CRN was established in 2013. It comprises modules for clinical diagnostics, biopsy and cytology tests, surgery, and the pathologist’s examination of the surgical specimens. The completeness of the diagnostic notifications has been over 90% and 100% since 2019 for diagnostic and surgical notifications, respectively. Data on smoking and other risk factors were not available [16].

### Variable definitions

The RML was grouped together with the upper lobes whenever comparing the upper and lower lobes. The histological groups including carcinoids and small-cell lung cancer (SCLC), were grouped together as neuroendocrine tumours (NETs). Information about the treatment modalities of surgery and radiotherapy was available. The variable “first treatment” was used to indicate which treatment was given first and within one year of diagnosis.

### Statistical methods

Standard statistics such as numbers, percentages and proportions were used. Pearson’s chi square test or t test was used when comparing groups, and Fisher’s exact test and test of proportions were used when comparing proportions. The proportion of tumours occurring within a particular part of the lung compared to the proportion of volume of that anatomical part was defined as the *relative proportion*. The volume of the different lung lobes was defined according to Yamada et al. [12]. For example, the relative proportion of tumours in the RUL is calculated as the proportion of tumours diagnosed in the RUL divided by the total volume the RUL makes of the lungs. To analyse whether the anatomical location of the tumour was associated with the chance of receiving surgery, logistic regressions were performed adjusting for age, sex, stage, histology, and EGFR-status. Uni-and multivariable Cox regressions were also performed to identify independent prognostic factors. The Cox regressions were additionally adjusted for type of first treatment. Likelihood ratio tests were used to determine the statistical significance of the covariates to be included in the final model. In the logistic and Cox regressions, multiple imputation was used to handle missing data on side and lobe, histology, cTNM and EGFR-results. The imputation model was run 30 times using the mi impute chained command in Stata [17]. In all other parts of this paper, the results are presented based on complete case data. All-cause (overall) median survival and relative survival [18, 19] were estimated for each patient characteristic. Relative survival was estimated using the stnet command in Stata [20]. Follow-up time was defined as the time from diagnosis to date of death or censoring, whichever occurred first. Complete vital information up until 31 December 2022 was available. A national population lifetable stratified by calendar year, sex and annual age was used to calculate expected survival. Relative survival was estimated using the Pohar Perme estimator [19], and here, the 95% CI was calculated using the delta method and a log–log transformation. A *p* value < 0.05 was considered significant. All analyses were performed in Stata.

## Results

### The study population

In the period 2018–2022, 17,410 patients were identified with a primary lung cancer diagnosis (ICD-10 code: C34) at the CRN. Of these, 24.9% (*n* = 4,335) were excluded since the examination notifications were missing or information on one or more of the following factors was missing: multidisciplinary team meeting (MDT), positron-emission tomography computer-tomography (PET-CT), endobronchial ultrasound fine needle aspiration cytology (EBUS), results from epidermal growth factor receptor (EGFR) analysis or staging by cTNM (*n* = 2,549). Patients with unknown morphology (*n* = 305) or side information (*n* = 468) were also excluded. Finally, we excluded patients with tumours in the main bronchus (*n* = 824) and those with either overlapping lesions or unknown tumour location (*n* = 627). As a result, 10,849 patients were eligible for analysis. The data by lobe are listed in Table 1. Tables with the same data but arranged by side (Supplementary Table 1) and by upper and lower lobes (Supplementary Table 2) are also presented. There was no indication of a shift in the distribution of tumours between the lung lobes during the period (data not shown).

**Table 1:** Patient characteristics per side and lobe among patients diagnosed with lung cancer in 2018–2022 in Norway

	Lobe											
	RUL		LUL		RML		RLL		LLL		Total	
**All**	3,381	(31.2%)	2,796	(25.8%)	587	(5.4%)	2,328	(21.5%)	1,757	(16.2%)	10,849	(100.0%)
**Age**	70.2	(8.9)	70.8	(8.9)	69.7	(10.2)	70.6	(9.6)	71.0	(9.3)	70.5	(9.2)
**Sex**												
Female	1,564	(46.3%)	1,325	(47.4%)	329	(56.0%)	1,187	(51.0%)	884	(50.3%)	5,289	(48.8%)
Male	1,817	(53.7%)	1,471	(52.6%)	258	(44.0%)	1,141	(49.0%)	873	(49.7%)	5,560	(51.2%)
**Histology**												
AC	1,796	(53.1%)	1,397	(50.0%)	287	(48.9%)	1,199	(51.5%)	899	(51.2%)	5,578	(51.4%)
SCC	752	(22.2%)	668	(23.9%)	99	(16.9%)	577	(24.8%)	427	(24.3%)	2,523	(23.3%)
NSCLC, NOS	270	(8.0%)	201	(7.2%)	27	(4.6%)	148	(6.4%)	95	(5.4%)	741	(6.8%)
Large-cell	45	(1.3%)	33	(1.2%)	8	(1.4%)	30	(1.3%)	20	(1.1%)	136	(1.3%)
SCLC	411	(12.2%)	398	(14.2%)	91	(15.5%)	270	(11.6%)	218	(12.4%)	1,388	(12.8%)
Carcinoid	44	(1.3%)	36	(1.3%)	62	(10.6%)	61	(2.6%)	65	(3.7%)	268	(2.5%)
Other	63	(1.9%)	63	(2.3%)	13	(2.2%)	43	(1.8%)	33	(1.9%)	215	(2.0%)
**cTNM**												
I	959	(28.4%)	768	(27.5%)	213	(36.3%)	716	(30.8%)	551	(31.4%)	3,207	(29.6%)
II	283	(8.4%)	233	(8.3%)	39	(6.6%)	222	(9.5%)	195	(11.1%)	972	(9.0%)
III	708	(20.9%)	539	(19.3%)	91	(15.5%)	434	(18.6%)	309	(17.6%)	2,081	(19.2%)
IV	1,431	(42.3%)	1,256	(44.9%)	244	(41.6%)	956	(41.1%)	702	(40.0%)	4,589	(42.3%)
**MDT**												
No	797	(23.6%)	664	(23.7%)	125	(21.3%)	554	(23.8%)	417	(23.7%)	2,557	(23.6%)
Yes	2,584	(76.4%)	2,132	(76.3%)	462	(78.7%)	1,774	(76.2%)	1,340	(76.3%)	8,292	(76.4%)
**PET-CT**												
No	1,174	(34.7%)	991	(35.4%)	204	(34.8%)	789	(33.9%)	585	(33.3%)	3,743	(34.5%)
Yes	2,207	(65.3%)	1,805	(64.6%)	383	(65.2%)	1,539	(66.1%)	1,172	(66.7%)	7,106	(65.5%)
**EBUS**												
No	2,419	(71.5%)	2,192	(78.4%)	430	(73.3%)	1,711	(73.5%)	1,342	(76.4%)	8,094	(74.6%)
Yes	962	(28.5%)	604	(21.6%)	157	(26.7%)	617	(26.5%)	415	(23.6%)	2,755	(25.4%)
**EGFR-test**												
Not tested	1,432	(42.4%)	1,316	(47.1%)	294	(50.1%)	1,068	(45.9%)	809	(46.0%)	4,919	(45.3%)
Positive	199	(5.9%)	170	(6.1%)	31	(5.3%)	111	(4.8%)	91	(5.2%)	602	(5.5%)
Negative	1,692	(50.0%)	1,255	(44.9%)	255	(43.4%)	1,118	(48.0%)	825	(47.0%)	5,145	(47.4%)
Unknown	58	(3.0%)	55	(3.7%)	7	(2.4%)	31	(2.5%)	32	(3.4%)	183	(3.1%)
**First treatment**												
Resected	871	(25.8%)	721	(25.8%)	196	(33.4%)	693	(29.8%)	532	(30.3%)	3,013	(27.8%)
SBRT	294	(8.7%)	211	(7.5%)	30	(5.1%)	197	(8.5%)	145	(8.3%)	877	(8.1%)
Curative RT	435	(12.9%)	337	(12.1%)	56	(9.5%)	227	(9.8%)	177	(10.1%)	1,232	(11.4%)
Palliative RT	703	(20.8%)	585	(20.9%)	90	(15.3%)	430	(18.5%)	313	(17.8%)	2,121	(19.6%)
Unknown RT	22	(0.7%)	19	(0.7%)	2	(0.3%)	9	(0.4%)	16	(0.9%)	68	(0.6%)
No treatment reported	1,056	(31.2%)	923	(33.0%)	213	(36.3%)	772	(33.2%)	574	(32.7%)	3,538	(32.6%)

Abbreviations: AC: adenocarcinoma, EBUS: endobronchial ultrasound fine needle aspiration cytology, EGFR: epidermal growth factor receptor, LLL: left lower lobe, LUL: left upper lobe, MDT: patients discussed in multidisciplinary team meeting, NSCLC NOS: non-small cell lung cancer not otherwise specified, RLL: right lower lobe, RML: right middle lobe, RT: radiotherapy, RUL: right upper lobe, SBRT: stereotactic body radiation therapy, SCC: squamous cell cancer, SCLC: small cell lung cancer

### Proportions of tumours

**Fig. 1:**
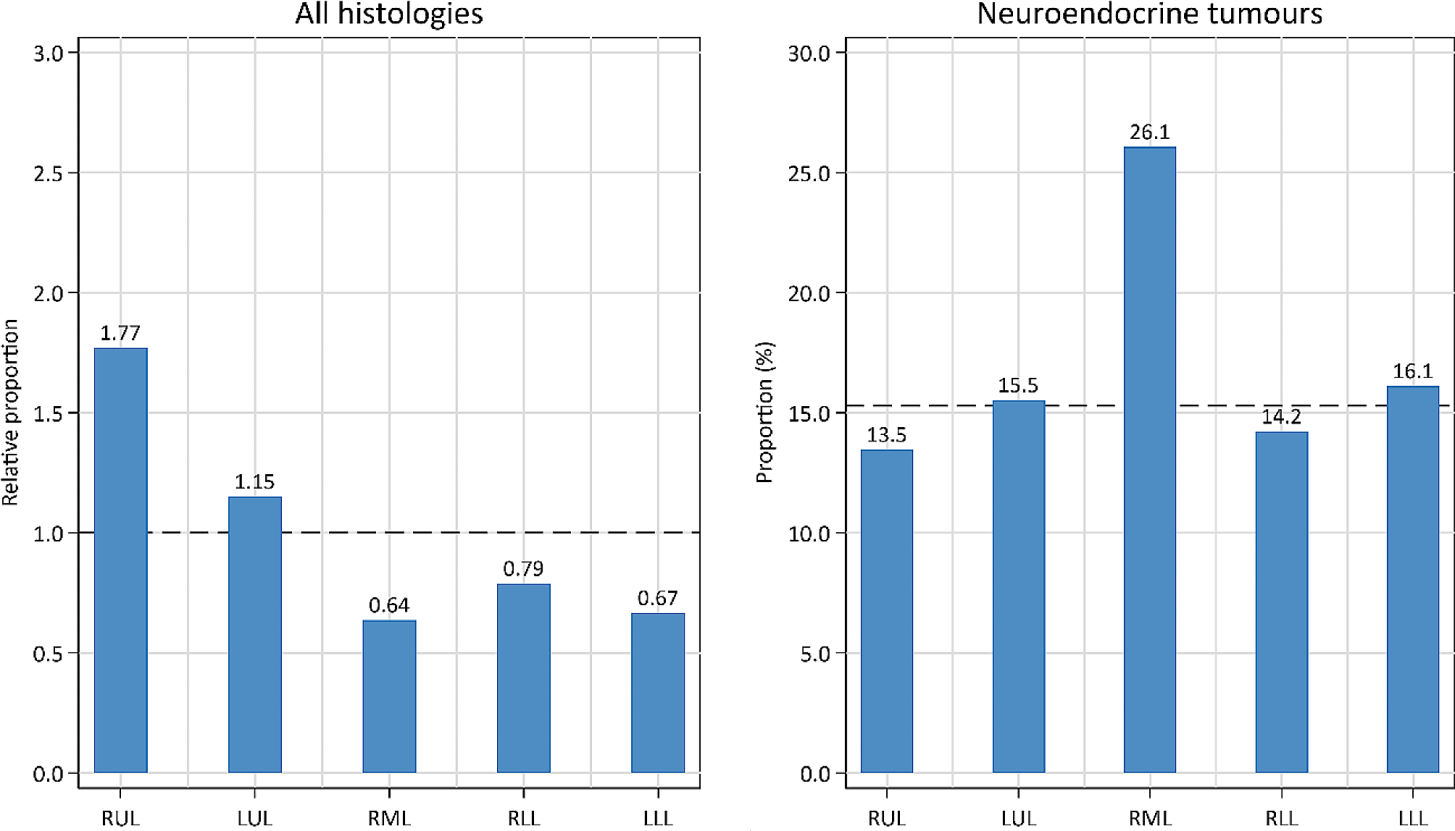
The relative proportion of all malignant tumours (left panel) and the proportion of the subgroup with neuroendocrine tumours by lung lobe (right panel) among patients diagnosed with lung cancer 2018–2022 in Norway. The dashed lines indicate the level if all tumours and subgroups were evenly distributed within the lung. Abbreviations: LLL: left lower lobe, LUL: left upper lobe, RLL: right lower lobe, RML: right middle lobe RUL: right upper lobe

The relative proportion of tumours in the lobes ranged from 0.64 in RML to 1.77 in the RUL (Fig. 1). The RUL has comparable volumes as the LUL, RLL and LLL, and the number of tumours in the RUL was increased (*p* < 0.001) compared to each of these lobes. In 62.3% (*n* = 6,764) of the patients, the tumour was in the upper lobes (Table 1), representing an increased relative proportion of 1.28 compared to the lower lobes with 0.73 (*p* < 0.001).

### Age

The mean age at diagnosis for all patients was 70.5 years (Table 1). Those with tumours on the right side had a mean age of 70.3 years and were younger than those with left-sided tumour (70.9 years, *p* < 0.001). Those with tumours in the upper lobes had a mean age of 70.4 years and were younger than those with tumours in the lower lobes (70.8 years, *p* < 0.028). Patients with tumours in the RML had the lowest mean age at 69.7 years, and those with tumours in the LLL had the highest mean age, 71.0 years (*p* = 0.002).

### Sex

Females comprised 48.8%. The proportion of females with tumours in the upper lobes was 47.6%, which is lower than the proportion in the lower lobes (50.7%, *p* = 0.002). The lobes with the lowest and highest proportions of females were the RUL and RML, with 46.3% and 56.0%, respectively (*p* < 0.001).

### Endobronchial ultrasound fine needle aspiration cytology

Endobronchial ultrasound fine needle aspiration cytology (EBUS) was performed in 25.4%, with 22.4% and 27.6% of patients with tumours in the left and right lung, respectively (*p* < 0.001).

### Epidermal growth factor receptor

The results from epidermal growth factor receptor (EGFR) mutation analyses were available in 87.8% (*n* = 4,897) of the patients with AC.

A positive EGFR mutation was reported in 12.5% (*n* = 379) of the patients with AC in the upper lobes and 10.5% (*n* = 196) in the lower lobes (*p* = 0.042). The lobes with the lowest and highest proportions of mutated EGFR were RLL and LUL, with 10.2% (*n* = 108) and 13.5% (*n* = 162) respectively (*p* = 0.013).

### Histology

AC was diagnosed in 51.4% of patients, and there were small differences in the proportions between the lung lobes (Table 1).

Squamous cell carcinoma (SCC) was diagnosed in 23.3%, and 22.5% of patients with tumours the upper lobes and 24.6% in the lower lobes (*p* = 0.011). SCC was found in 16.9% of the RMLs compared to 23.6% in the four other lobes combined (*p* < 0.001).

Small cell lung cancer (SCLC) was diagnosed in 12.8%, and 11.9% of patients, with tumours in the lower lobes and 13.3% in the upper lobes (*p* = 0.040). The lobes with the lowest and highest proportions of SCLC were RLL and RML with 11.6% and 15.5%, respectively (*p* = 0.010).

Carcinoid tumours were diagnosed in 2.5%, and 2.1% of patients with tumours in the upper lobes and 3.1% in the lower lobes (*p* = 0.001). The lobes with the lowest and highest proportions of carcinoid tumours were LUL and RML with 1.3% and 10.6%, respectively (*p* < 0.001). Of all carcinoid tumours reported, 23.1% (*n* = 62) were in the RML.

Due to their common cellular origin, SCLC and carcinoids are grouped as neuroendocrine tumours (NETs) and were diagnosed in 15.3%, with 26.1% in the RML and 14.6% in the rest of the lung (*p* < 0.001) (Fig. 1).

### Tumour diameter, lymph node metastases, and stage

The mean tumour diameter was 43.9 (SD = 32.4) mm, with the smallest at 38.0 (SD = 25.2) mm in RML and the largest at 45.6 (SD = 38.8) mm in LUL (*p* < 0.001).

Metastases to mediastinal lymph nodes (cN2 or cN3) were reported in 43.6% (*n* = 4,693) of the patients. The lowest proportion of positive cN2 or cN3 nodes was reported in LLL with 37.8% (*n* = 658) and the highest in RUL with 46.7% (*n* = 1,571) (*p* < 0.001). The proportions of tumours with mediastinal lymph node metastases in the upper and lower lobes were 45.8% (*n* = 3,074) and 40.1% (*n* = 1,619), respectively (*p* < 0.001). In the right and left lungs there were 44.6% (*n* = 2,782) and 42.3% (*n* = 1,911) mediastinal nodes, respectively (*p* = 0.02).

The proportions diagnosed in cTNM- stages I, II, III and IV were 29.6%, 9.0%, 19.2%, and 42.3% respectively (Table 1). In patients with tumours in the upper and lower lobes, the proportions of patients with stage I and II disease were 36.9% (*n* = 2495) and 41.2% (*n* = 1,684), respectively (*p* < 0.001).

The lobes with the lowest and highest proportions of patients diagnosed in stages I and II were LUL and RML, with 35.8% and 42.9%, respectively (*p* = 0.001).

### Treatment

Surgical resection was performed in 27.8%, and 30.4% of patients with tumours in the upper and lower lobes, respectively (*p* < 0.001). The highest resection rate was 33.4% in the RML, while 27.5% of the patients with tumours in the other four lobes were resected (*p* = 0.002). Both by uni- and multivariable regression analysis, tumour in the RLL was a significant predictor for resection.

Stereotactic body radiation therapy (SBRT), or curative radiotherapy, was given to 20.1% of the patients with tumours in the upper lobes and 18.3% in the lower lobes (*p* = 0.016). Palliative radiotherapy was performed in 1378 (20.4%) in the upper- and in 743 (18.2%) in the lower lobes (*p* = 0.005). Lobe, sex, age, cTNM stage, histologic type, and EGFR status were found to be independent predictors for resection (Table 2).

**Table 2:** The probability of having a surgical resection of a malign lung tumour, logistic regression for patients diagnosed with lung cancer in 2018–2022 in Norway

	Univariable	Multivariable
	Odds Ratio (95%CI)	Odds Ratio (95%CI)
Lobe		
RUL	1.00	1.00
LUL	0.98 (0.88–1.09)	1.01 (0.88–1.16)
RML	1.40 (1.17–1.67)	1.19 (0.93–1.54)
RLL	1.22 (1.10–1.36)	1.21 (1.06–1.41)
LLL	1.19 (1.06–1.34)	1.14 (0.97–1.34)
*p*-value	0.00	0.04
Sex		
Female	1.00	1.00
Male	0.84 (0.78–0.90)	1.08 (0.98–1.20)
*p*-value	0.00	0.13
Age	0.96 (0.95–0.96)	0.92 (0.92–0.93)
*p*-value	0.00	0.00
cTNM		
I	1.00	1.00
II	0.91 (0.80–1.04)	1.15 (0.99–1.33)
III	0.13 (0.12–0.15)	0.14 (0.12–0.16)
IV	0.01 (0.01–0.01)	0.01 (0.01–0.01)
*p*-value	0.00	0.00
Histology		
AC	1.00	1.00
SCC	0.79 (0.72–0.87)	0.66 (0.58–0.74)
NSCLC, NOS	0.11 (0.08–0.15)	0.17 (0.11–0.24)
Large-cell	1.24 (0.91–1.70)	2.10 (1.32–3.35)
SCLC	0.06 (0.04–0.08)	0.14 (0.11–0.20)
Carcinoid	6,36 (4.96–8.16)	1.57 (1.16–2.12)
Other	2.03 (1.60–2.58)	2.49 (1.72–3.59)
*p*-value	0.00	0.00
EGFR		
Not tested		1.58 (1.22–2.05)
Positive		1.00
Negative		3.01 (2.51–3.62)
*p*-value		0.00

Abbreviations: AC: adenocarcinoma, CI: confidence interval, EGFR: epidermal growth factor receptor, LLL: left lower lobe, LUL: left upper lobe, NSCLC NOS: non-small cell lung cancer not otherwise specified, RLL: right lower lobe, RML: right middle lobe, RUL: right upper lobe, SCC: squamous cell cancer, SCLC: small cell lung cancer

### Survival

The 5-year relative survival in the study group was 33.7% (Table 3). The numerically reduced survival in patients with tumours in the LUL (Table 3) was not significant in multivariable analysis (Table 4). Independent predictors for survival in multivariable analysis were age, sex, cTNM stage, histology, EGFR status, and type of treatment (*p* < 0.001). The location in the lung lobes was not a significant predictor of survival.

**Table 3:** 1-year, 5-year relative survival, and median survival by groups for patients diagnosed with lung cancer in 2018–2022 in Norway

	1-year RS (95%CI)	5-year RS (95%CI)	Median survival (95%CI)
All	61.2 (60.4–62.1)	33.7 (32.7–34.7)	20.1 (19.1–21.0)
Side			
Right	61.2 (60.1–62.4)	34.6 (33.2–35.9)	20.6 (19.1–22.0)
Left	61.2 (59.9–62.5)	32.5 (31.0-34.1)	19.6 (18.4–20.8)
Lobe			
RUL	61.1 (59.6–62.7)	34.1 (32.3–35.9)	20.8 (18.5–22.6)
LUL	60.4 (58.7–62.1)	31.6 (29.8–33.6)	18.8 (17.1–20.3)
RML	62.7 (59.0-66.6)	36.5 (32.0-41.5)	20.4 (16.3–25.5)
RLL	61.2 (59.3–63.1)	34.9 (32.7–37.2)	20.5 (18.6–22.6)
LLL	62.5 (60.4–64.7)	33.9 (31.5–36.5)	20.8 (18.8–23.0)
Upper lobes / Lower lobes			
Upper	60.8 (59.7–61.9)	33.0 (31.7–34.3)	19.5 (18.4–20.9)
Lower	61.9 (60.6–63.2)	34.7 (33.2–36.4)	20.7 (19.1–22.0)
Sex			
Female	65.3 (64.1–66.5)	37.7 (36.3–39.2)	25.3 (23.7–27.0)
Male	57.3 (56.1–58.5)	29.7 (28.4–31.1)	15.9 (14.9–16.8)
cTNM			
I	93.4 (92.4–94.4)	68.5 (66.1–71.0)	82.7 (77.3–87.3)
II	82.6 (80.2–85.1)	49.9 (46.0-54.2)	47.0 (40.8–53.4)
III	65.2 (63.1–67.2)	25.2 (23.0-27.6)	18.9 (17.8–20.1)
IV	31.4 (30.1–32.7)	7.8 (6.8-9.0)	6.1 (5.7–6.4)
Unknown	57.0 (51.2–63.4)	27.1 (21.6–34.0)	12.0 (9.3–15.6)
Histology			
AC	69.4 (68.2–70.7)	41.5 (39.9–43.3)	31.3 (29.4–33.6)
SCC	64.2 (62.3–66.1)	34.8 (32.7–37.0)	21.5 (19.3–23.5)
NSCLC, NOS	42.4 (39.0-46.1)	18.4 (15.2–22.1)	7.8 (6.9–9.5)
Large-cell	49.6 (41.4–59.3)	25.8 (19.2–34.7)	12.0 (7.8–17.6)
SCLC	36.9 (34.5–39.6)	8.9 (7.4–10.7)	8.3 (7.7-9.0)
Carcinoid	97.9 (95.0-100.9)	90.7 (82.1-100.1)	NA
Other	60.3 (53.5–68.0)	34.7 (26.4–45.7)	27.4 (16.3–38.0)
EGFR			
Not tested	59.9 (55.8–64.2)	43.6 (39.3–48.3)	15.3 (14.1–16.2)
Positive	84.4 (81.3–87.7)	46.8 (41.1–53.2)	42.4 (36.0-49.4)
Negative	69.0 (67.6–70.5)	40.2 (38.2–42.2)	26.8 (25.4–28.8)
Unknown	67.9 (60.0-76.8)	44.6 (36.0-55.4)	30.4 (17.4–44.5)

Abbreviations: AC: adenocarcinoma, CI: confidence interval, EGFR: epidermal growth factor receptor, LLL: left lower lobe, LUL: left upper lobe, NA: not available, NSCLC NOS: non-small cell lung cancer not otherwise specified, RLL: right lower lobe, RML: right middle lobe, RS: relative survival, RUL: right upper lobe, SCC: squamous cell cancer, SCLC: small cell lung cancer

**Table 4:** The risk of death for lung cancer patients, Cox regression for patients diagnosed with lung cancer in 2018–2022 in Norway

	Univariable	Multivariable
	Hazard Ratio (95%CI)	Hazard Ratio (95%CI)
Lobe		
RUL	1.00	1.00
LUL	1.04 (0.98–1.10)	1.01 (0.95–1.07)
RML	0.97 (0.87–1.09)	1.09 (0.97–1.22)
RLL	0.99 (0.92–1.05)	0.99 (0.92–1.06)
LLL	0.99 (0.92–1.07)	1.02 (0.95–1.10)
*p*-value	0.55	0.56
Age	1.03 (1.03–1.03)	1.03 (1.02–1.03)
*p*-value	0.00	0.00
Sex		
Female	1.00	1.00
Male	1.28 (1.23–1.34)	1.21 (1.16–1.27)
*p*-value	0.00	0.00
cTNM		
I	1.00	1.00
II	1.94 (1.73–2.18)	1.48 (1.31–1.68)
III	3.72 (3.41–4.05)	1.98 (1.79–2.19)
IV	8.74 (8.11–9.42)	3.46 (3.13–3.81)
*p*-value	0.00	0.00
Histology		
AC	1.00	1.00
SCC	1.24 (1.17–1.31)	1.40 (1.32–1.49)
NSCLC- NOS	2.05 (1.89–2.23)	1.44 (1.33–1.57)
Large-cell	1.50 (1.24–1.80)	1.54 (1.28–1.86)
SCLC	2.46 (2.32–2.60)	1.62 (1.52–1.71)
Carcinoid	0.14 (0.10–0.20)	0.35 (0.25–0.49)
Other	1.13 (0.96–1.32)	1.45 (1.23–1.70)
*p*-value	0.00	0.00
EGFR		
Not tested		1.50 (1.33–1.69)
Positive		1.00
Negative		0.93 (0.55–1.02)
*p*-value		0.00
First treatment		
Resected	1.00	1.00
SBRT	2.07 (1.82–2.35)	1.74 (1.52–1.98)
Cur rad	3.73 (3.34–4.17)	1.95 (1.73–2.20)
Pall rad	11.05 (10.05–12.15)	4.14 (3.70–4.63)
Unknown rad	8.51 (6.85–10.57)	2.94 (2.34–3.69)
No treatment reported	12.50 (11.42–13.69)	4.97 (4.47–5.54)
*p*-value	0.00	0.00

Abbreviations: AC: adenocarcinoma, CI: confidence interval, LLL: left lower lobe, LUL: left upper lobe, NSCLC NOS: non-small cell lung cancer not otherwise specified, RLL: right lower lobe, RML: right middle lobe, RUL: right upper lobe, SBRT: stereotactic body radiation therapy, SCC: squamous cell cancer, SCLC: small cell lung cancer

## Discussion

This study showed considerable differences in the proportion and histological types of malignant tumours between the lung lobes, with the most pronounced increased relative proportion of tumours in the RUL. There were also differences in the histologic types where the high relative proportion of NETs in the RML was the most noteworthy. There were also differences in the diagnostics and treatment modalities between the lobes. The location of the tumour did not predict survival.

An even more pronounced difference in the proportion of tumours between the RUL and LLL was published in 1949 [21]. The skewed distribution of lung tumours found in the present study is comparable with more recent data from others who emphasized other topics [3, 6, 7]. Our finding of an increased proportion of patients with AC and EGFR-positive mutations in the upper lobes is consistent with previous reports [8, 9]. Further, Hill et al. have recently described the significance of air pollutants in inducing EGFR-driven ACs in never smokers [22].

The present finding of improved survival in females compared to men is consistent with data in previous reports [23, 24]. However, due to the exclusion of patients with tumours in the main bronchi and overlapping or unknown tumour locations, the present data on survival, staging, and treatment modalities are not representative for comparison with data from groups with unselected patients.

The increased proportion of surgery performed when tumours are in the lower lobes is probably due to tumours here are diagnosed in earlier stage and with fewer positive mediastinal lymph nodes than the tumours in the upper lobes. The reason for earlier stage and less positive mediastinal lymph nodes in tumours in the lower lobes is unclear but a possible increased sensitivity for chest x-ray and CT-scan when tumours are in the lower lobes may play a role.

Limitations of this study are the lack of information on smoking, performance- and socioeconomic status and that it is retrospective. Furthermore, the reference material from Yamada et al. used for the proportion of the volumes of the lobes is based on a limited number of healthy volunteers in another population than the Nordic. However, the variation in their data is low [12], and the differences found here are pronounced, thus, the risk of erroneous conclusions seems to be small. The strength of this study is the completeness of national data.

Most deviations from the rest of the lobes were found for the RML, which had the lowest relative proportion of tumours, highest proportion of females, the smallest tumours, the lowest cTNM stage, the lowest age, and the lowest proportion of SCC and NSCLC NOS. The neuroendocrine tumours – the SCLC and the carcinoids, representing the poorest and best prognosis of the lung tumours, respectively both had their highest proportion in the RML. This cannot be explained by respiratory pattern alone, and biological factors must also be of importance and would need further studies to elucidate.

Despite the embryological and anatomical relation between the RUL and the RML, the two lobes differ markedly in many of the present findings. In comparing the upper and lower parts of the lung, the RML has in part been included in the upper [5, 7, 9] or the lower lobes [6], and this may give conflicting results in comparing what has been defined as the upper and lower parts of the lung.

A possible theory of a common pathogenetic factor may be that the increased proportion of malignant tumours in the upper and right lung is equivalent to the predilection of tuberculosis and pneumoconiosis [13, 25–29]. All three diseases are caused by inhaled pathogenic substances – bacilli, inorganic particles, and tobacco smoke. Thus, the pattern of respiration and the anatomy of the large airways may be common factors. The tendency of inhaled substances to affect the upper lobes is in line with the findings of Milic-Emili et al. that ventilation at rest and mild exercise are mainly performed in the upper parts, whereas at increased physical activity, the entire lung is activated [30]. The propensity for the right side may be explained by “Slightly more dust is deposited in the right lung than the left, probably because the right main bronchus is more in line with the trachea, and is broader and shorter than the left, and carries 55% of the inhaled air” [13]. The increased proportion of SCLC, the tumour with the strongest correlation to tobacco smoke [31], in the upper lobes may support the view that these lobes are most exposed to the inhaled smoke. Additionally, the reduced age in those with tumours in the upper lobes and right lung may be explained by increased exposure to tobacco inducing malignancy at a younger age.

The data in the present study showed marked differences in the proportion and characteristics of malignant tumours by lung lobe. Particularly, the increase in the proportion of malignant tumours in the right upper lobe is similar to the reported preponderance for tuberculosis and pneumoconiosis in the same lobe may reflect a common pathogenesis caused by the respiratory pattern and the airway anatomy, which gives rise to an increase in bacilli, inorganic dust particles and carcinogens to the upper and right part of the lungs.

### Electronic supplementary material

Below is the link to the electronic supplementary material.

Supplementary Material 1

Supplementary Material 2

## Data Availability

Data underlying this article can be requested from the Cancer Registry of Norway through https://helsedata.no.

## Declarations

### Declarations

This study has been carried out in accordance with the guidelines in Declaration of Helsinki.

## Abbreviations

AC: Adenocarcinoma
CI: Confidence interval
CRN: Cancer Registry of Norway
EBUS: Endobronchial ultrasound fine needle aspiration cytology
EGFR: Epidermal growth factor receptor
LLL: Left lower lobe
LUL: Left upper lobe
MDT: Multi-disciplinary team meeting
NET: Neuroendocrine tumour
NSCLC NOS: Non-small cell lung cancer not otherwise specified
PET-CT: Positron-emission tomography computer-tomography
RLL: Right lower lobe
RML: Right middle lobe
RS: Relative survival
RUL: Right upper lobe
SBRT: Stereotactic body radiation therapy
SCC: Squamous cell carcinoma
SCLC: Small cell lung cancer
SD: Standard deviation
