# Supplementary material for: Distribution and characteristics of malignant tumours by lung lobe
Source: BMC Pulm Med. 2024 Mar 4;24:106. doi: 10.1186/s12890-024-02918-w (PMC10910834; doi:10.1186/s12890-024-02918-w)
Supplement: Supplementary file 1 — Supplementary Material 1 [file 12890_2024_2918_MOESM1_ESM.docx]

| Table ad1: Patient characteristics per side among patients diagnosed with lung cancer in 2018-2022 in Norway | | | | | | | |
| --- | --- | --- | --- | --- | --- | --- | --- |
|  | Side | | | | | | |
|  | Right | | | Left | | Total | |
| Age | 70.3 | | (9.3) | 70.9 | (9.0) | 70.5 | (9.2) |
|  |  | |  |  |  |  |  |
| Sex |  | |  |  |  |  |  |
| Female | 3,080 | | (48.9%) | 2,209 | (48.5%) | 5,289 | (48.8%) |
| Male | 3,216 | | (51.1%) | 2,344 | (51.5%) | 5,560 | (51.2%) |
|  |  | |  |  |  |  |  |
| Morphology |  | |  |  |  |  |  |
| AC | 3,282 | | (52.1%) | 2,296 | (50.4%) | 5,578 | (51.4%) |
| SCC | 1,428 | | (22.7%) | 1,095 | (24.1%) | 2,523 | (23.3%) |
| NSCLC, NOS | 445 | | (7.1%) | 296 | (6.5%) | 741 | (6.8%) |
| Large-cell | 83 | | (1.3%) | 53 | (1.2%) | 136 | (1.3%) |
| SCLC | 772 | | (12.3%) | 616 | (13.5%) | 1,388 | (12.8%) |
| Carcinoid | 167 | | (2.7%) | 101 | (2.2%) | 268 | (2.5%) |
| Other | 119 | | (1.9%) | 96 | (2.1%) | 215 | (2.0%) |
|  |  | |  |  |  |  |  |
| cTNM |  | |  |  |  |  |  |
| I | 1,888 | | (30.0%) | 1,319 | (29.0%) | 3,207 | (29.6%) |
| II | 544 | | (8.6%) | 428 | (9.4%) | 972 | (9.0%) |
| III | 1,233 | | (19.6%) | 848 | (18.6%) | 2,081 | (19.2%) |
| IV | 2,631 | | (41.8%) | 1,958 | (43.0%) | 4,589 | (42.3%) |
|  |  | |  |  |  |  |  |
| MDT |  | |  |  |  |  |  |
| No | 1,476 | | (23.4%) | 1,081 | (23.7%) | 2,557 | (23.6%) |
| Yes | 4,820 | | (76.6%) | 3,472 | (76.3%) | 8,292 | (76.4%) |
|  |  | |  |  |  |  |  |
| PET-CT |  | |  |  |  |  |  |
| No | 2,167 | | (34.4%) | 1,576 | (34.6%) | 3,743 | (34.5%) |
| Yes | 4,129 | | (65.6%) | 2,977 | (65.4%) | 7,106 | (65.5%) |
|  |  | |  |  |  |  |  |
| EBUS |  | |  |  |  |  |  |
| No | 4,560 | | (72.4%) | 3,534 | (77.6%) | 8,094 | (74.6%) |
| Yes | 1,736 | | (27.6%) | 1,019 | (22.4%) | 2,755 | (25.4%) |
|  |  | |  |  |  |  |  |
| EGFR-test |  | |  |  |  |  |  |
| No | 2,794 | | (44.4%) | 2,125 | (46.7%) | 4,919 | (45.3%) |
| Yes | 3,502 | | (55.6%) | 2,428 | (53.3%) | 5,930 | (54.7%) |
|  |  | |  |  |  |  |  |
| EGFR-result |  | |  |  |  |  |  |
| Positive | 341 | | (5.4%) | 261 | (5.7%) | 602 | (5.5%) |
| Negative | 3,065 | | (48.7%) | 2,080 | (45.7%) | 5,145 | (47.4%) |
| Unknown | 2,890 | | (45.9%) | 2,212 | (48.6%) | 5,102 | (47.0%) |
|  |  | |  |  |  |  |  |
| First treatment | |  |  |  |  |  |  |
| Resected | 1,760 | | (28.0%) | 1,253 | (27.5%) | 3,013 | (27.8%) |
| SBRT | 521 | | (8.3%) | 356 | (7.8%) | 877 | (8.1%) |
| Cur rad | 718 | | (11.4%) | 514 | (11.3%) | 1,232 | (11.4%) |
| Pall rad | 1,223 | | (19.4%) | 898 | (19.7%) | 2,121 | (19.6%) |
| Unknown rad | 33 | | (0.5%) | 35 | (0.8%) | 68 | (0.6%) |
| No TX | 2,041 | | (32.4%) | 1,497 | (32.9%) | 3,538 | (32.6%) |

Abbreviations: AC: adenocarcinoma, EBUS: endobronchial ultrasound fine needle aspiration cytology, EGFR: epidermal growth factor receptor, LLL: left lower lobe, LUL: left upper lobe, MDT: patients discussed in multidisciplinary team meeting, NSCLC NOS: non-small cell lung cancer not otherwise specified, No TX: no treatment reported, RLL: right lower lobe, RML: right middle lobe, RUL: right upper lobe, SBRT: stereotactic body radiation therapy, SCC: squamous cell cancer, SCLC: small cell lung cancer.
