# Supplementary material for: Distribution and characteristics of malignant tumours by lung lobe
Source: BMC Pulm Med. 2024 Mar 4;24:106. doi: 10.1186/s12890-024-02918-w (PMC10910834; doi:10.1186/s12890-024-02918-w)
Supplement: Supplementary file 2 — Supplementary Material 2 [file 12890_2024_2918_MOESM2_ESM.docx]

| Table ad2: Patient characteristics per upper and lower lobes among patients diagnosed with lung cancer in 2018-2022 in Norway | | | | | | |
| --- | --- | --- | --- | --- | --- | --- |
|  | Upper vs lower lobes | | | | | |
|  | Upper | | Lower | | Total | |
| Age | 70.4 | (9.0) | 70.8 | (9.5) | 70.5 | (9.2) |
|  |  |  |  |  |  |  |
| Sex |  |  |  |  |  |  |
| Female | 3,218 | (47.6%) | 2,071 | (50.7%) | 5,289 | (48.8%) |
| Male | 3,546 | (52.4%) | 2,014 | (49.3%) | 5,560 | (51.2%) |
|  |  |  |  |  |  |  |
| Morphology |  |  |  |  |  |  |
| Adeno | 3,480 | (51.4%) | 2,098 | (51.4%) | 5,578 | (51.4%) |
| SCC | 1,519 | (22.5%) | 1,004 | (24.6%) | 2,523 | (23.3%) |
| NSCLC, NOS | 498 | (7.4%) | 243 | (5.9%) | 741 | (6.8%) |
| Large-cell | 86 | (1.3%) | 50 | (1.2%) | 136 | (1.3%) |
| SCLC | 900 | (13.3%) | 488 | (11.9%) | 1,388 | (12.8%) |
| Carcinoid | 142 | (2.1%) | 126 | (3.1%) | 268 | (2.5%) |
| Other | 139 | (2.1%) | 76 | (1.9%) | 215 | (2.0%) |
|  |  |  |  |  |  |  |
| cTNM |  |  |  |  |  |  |
| I | 1,940 | (28.7%) | 1,267 | (31.0%) | 3,207 | (29.6%) |
| II | 555 | (8.2%) | 417 | (10.2%) | 972 | (9.0%) |
| III | 1,338 | (19.8%) | 743 | (18.2%) | 2,081 | (19.2%) |
| IV | 2,931 | (43.3%) | 1,658 | (40.6%) | 4,589 | (42.3%) |
|  |  |  |  |  |  |  |
| MDT |  |  |  |  |  |  |
| No | 1,586 | (23.4%) | 971 | (23.8%) | 2,557 | (23.6%) |
| Yes | 5,178 | (76.6%) | 3,114 | (76.2%) | 8,292 | (76.4%) |
|  |  |  |  |  |  |  |
| PET-CT |  |  |  |  |  |  |
| No | 2,369 | (35.0%) | 1,374 | (33.6%) | 3,743 | (34.5%) |
| Yes | 4,395 | (65.0%) | 2,711 | (66.4%) | 7,106 | (65.5%) |
|  |  |  |  |  |  |  |
| EBUS |  |  |  |  |  |  |
| No | 5,041 | (74.5%) | 3,053 | (74.7%) | 8,094 | (74.6%) |
| Yes | 1,723 | (25.5%) | 1,032 | (25.3%) | 2,755 | (25.4%) |
|  |  |  |  |  |  |  |
| EGFR-test |  |  |  |  |  |  |
| No | 3,042 | (45.0%) | 1,877 | (45.9%) | 4,919 | (45.3%) |
| Yes | 3,722 | (55.0%) | 2,208 | (54.1%) | 5,930 | (54.7%) |
|  |  |  |  |  |  |  |
| EGFR-result |  |  |  |  |  |  |
| Positive | 400 | (5.9%) | 202 | (4.9%) | 602 | (5.5%) |
| Negative | 3,202 | (47.3%) | 1,943 | (47.6%) | 5,145 | (47.4%) |
| Unknown | 3,162 | (46.7%) | 1,940 | (47.5%) | 5,102 | (47.0%) |
|  |  |  |  |  |  |  |
| First treatment |  |  |  |  |  |  |
| Resected | 1,788 | (26.4%) | 1,225 | (30.0%) | 3,013 | (27.8%) |
| SBRT | 535 | (7.9%) | 342 | (8.4%) | 877 | (8.1%) |
| Cur rad | 828 | (12.2%) | 404 | (9.9%) | 1,232 | (11.4%) |
| Pall rad | 1,378 | (20.4%) | 743 | (18.2%) | 2,121 | (19.6%) |
| Unknown rad | 43 | (0.6%) | 25 | (0.6%) | 68 | (0.6%) |
| No TX | 2,192 | (32.4%) | 1,346 | (32.9%) | 3,538 | (32.6%) |

Abbreviations: AC: adenocarcinoma, EBUS: endobronchial ultrasound fine needle aspiration cytology, EGFR: epidermal growth factor receptor, LLL: left lower lobe, LUL: left upper lobe, MDT: patients discussed in multidisciplinary team meeting, NSCLC NOS: non-small cell lung cancer not otherwise specified, No TX: no treatment reported, RLL: right lower lobe, RML: right middle lobe, RUL: right upper lobe, SBRT: stereotactic body radiation therapy, SCC: squamous cell cancer, SCLC: small cell lung cancer.
